# *In situ* constructed zeolite membranes on rough supports with the assistance of reticulated hydrotalcite interlayer†

**DOI:** 10.1039/d1ra05132f

**Published:** 2021-11-17

**Authors:** Wen-Yan Pan, Liang-Liang Peng, Wen-Jing Wang, Yuan-Yuan Li, Xue-Ling Wei

**Affiliations:** School of Chemical and Environmental Engineering, Anhui Polytechnic University Wuhu Anhui 241000 China xueling_wei@163.com

## Abstract

Zeolite membranes with unique physical and chemical properties are emerging as attractive candidates for membrane separation. However, defects in the zeolite layer seriously affect their molecular sieving performance. In this study, a novel strategy for preparing compact zeolite membranes on rough supports with the assistance of a reticulated hydrotalcite layer was developed. The reticulated hydrotalcite layer was grown on the inner surface of a 170 mm length ceramic tube by an *in situ* hydrothermal method, and a NaA zeolite membrane was prepared on this reticulated layer by the microwave-heating method. The hydrotalcite interlayer could not only improve the smoothness and regularity of the surface of the support but also fix the Si/Al active ingredients using its reticulate structure, finally effectively improving the quality and stability of the zeolite layer. The optimal molar ratio of the synthesis solution for the synthesis of the zeolite membrane was 3Na_2_O : 2SiO_2_ : Al_2_O_3_ : 200H_2_O. The permeance flux of H_2_ through the zeolite membrane synthesized under the optimal conditions was high as 0.47 × 10^−6^ mol m^−2^ s^−1^ Pa^−1^, and its permselectivity for H_2_ over N_2_ was 4.7, which was higher than the corresponding Knudsen diffusion coefficient. This study provides a new idea for the preparation of defect-free membranes on rough supports.

## Abstract 2

Reticulated hydrotalcite interlayer controls infiltration of active ingredients into the support, improving the quality and stability of the zeolite membrane.

## Graphical abstract

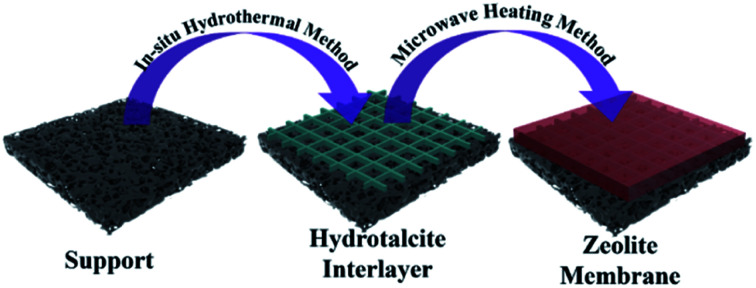

## 1. Introduction

Membrane separation has received great attention over the past decades.^1^ Zeolite membranes, considered attractive potential materials for membrane separation, can provide extremely high permeability and selectivity with excellent stability for the permeation separation of mixtures due to their unique physical and chemical properties.^2^ The preparation technology and quality of zeolite membranes have been greatly improved since their first preparation in the 1980s.^3^ Currently, zeolite membranes have been successfully applied in the field of pervaporation; however, they usually contain many defects larger than the pore size of the zeolite crystal. These defects usually provide nonselective pathways and thus worsen the inherent molecular-sieving-based separation performance.^4^ Therefore, the most important requirement for the industrial application of zeolite membranes is the elimination of defects in the membrane.

In order to eliminate defects and improve the quality of the membrane, various membrane preparation methods^5–8^ and post-treatment techniques^9–12^ have been developed to synthesize and/or modify zeolite membranes. Recently, Wei *et al.* eliminated the defects caused by bubbles by pre-degassing the synthesis system before the pressure-driven hydrothermal synthesis,^13^ and then modified the zeolite membranes with the help of the space network structure formed by methylcellulose at high temperature.^14^ Like other preparation methods and post-treatment techniques, those methods improved the density of the zeolite membrane to a certain extent, but it is still difficult to eliminate the defects completely so as to provide satisfactory permeation performance with excellent reproducibility in zeolite membranes. Reportedly, the rough and porous support results in uncontrolled infiltration of the active ingredients into the surface of the support, leading to the formation of defects in the zeolite layer and reducing the density of the zeolite membrane.^15,16^ Therefore, making the active ingredients evenly distributed on the rough surface of the support is the key to the preparation of compact zeolite membranes.

In recent years, researchers assembled organics, such as cationic polymers^17,18^ or covalent linkers,^19,20^ on the rough surface of the support and used the organics to capture the active ingredients in the synthesis solution, promoting the formation of well intergrown and compact zeolite membranes. While this method could provide appreciably high selectivity membranes, the membranes usually exhibited low permeability with stability issues owing to the organics contained in the membrane. Furthermore, the synthesis process lacks environmental benignity due to the employment of organic agents. Another common method to promote the compactness of the zeolite membrane is using smooth support to replace the rough support.^21,22^ The smooth support is composed of nanoparticles or coated with nanoparticles on the rough surface. While smooth support was helpful in preparing a high-quality zeolite membrane, it would increase the membrane preparation cost and thus limit its industrial application.^23^

In this work, a novel strategy for the preparation of compact zeolite membrane on rough tube support using a microwave heating method with the assistance of a reticulated hydrotalcite interlayer is reported. The grid-like reticulated hydrotalcite layer was *in situ* grown on the surface of the rough tube support and then NaA zeolite membrane was grown on this interlayer by the microwave heating method. The characterization results showed that the hydrotalcite interlayer can not only improve the flatness and integrity of the support, limiting the formation of defects but also can control the infiltration of the active ingredients onto the surface of support using its reticulated structure, improving the stability of the top layer with the bottom layer. This work is helpful for the preparation of defect-free membranes on rough support.

## 2. Experimental

### 2.1 Materials

All chemicals, including MgCl_2_·6H_2_O (AR, 99%), Al(NO_3_)_3_·9H_2_O (AR, 99%), Na_2_CO_3_·10H_2_O (AR, 99%), NaOH (AR, 96%), NaAlO_2_ (AR, 98%), Na_2_SiO_3_·9H_2_O (AR, 98%) were purchased from Shanghai Aladdin Biochemical Technology Co., Ltd., without further purification.

Porous α-A1_2_O_3_ tubes (outside diameter of 12 mm, inside diameter of 8 mm, length of 170 mm, the porosity of 50%, and average pore size of 1 μm) were purchased from Ningbo Damo Advanced Materials Technology Co., Ltd. and were used as support. The inner surface of the support was polished with 400 and 1000 grit-sand paper before using as support. Briefly, the sandpaper was cut into strips with a width of about 1 cm and wrapped around the glass rod fixing with double-sided tape. The glass rod was inserted into the ceramic tube and rotated in the same direction. When the surface of the support becomes smooth, the rotation of the glass rod could be stopped.

After polishing with sandpaper, the tubes were soaked in 12 mol L^−1^ sodium hydroxide solution for 12 h and then washed with deionized water repeatedly in an ultrasonic cleaner to remove the impurities after pretreatment. Finally, tubes were dried at 110 °C before further treatment.

### 2.2 Preparation of reticulated hydrotalcite layer

The hydrotalcite layer was synthesized by the *in situ* hydrothermal heating method. The influencing factors, including alkalinity of synthesis sol, Mg/Al ratio, and synthesis temperature, for the growth of the hydrotalcite layer, were investigated, and the results are shown in Fig. S1–S3.† The optimized synthesis conditions were as follows: synthesis solution molar composition 6MgCl_2_·6H_2_O : 2Al(NO_3_)_3_·9H_2_O : 20NaOH : 1Na_2_CO_3_ : 666H_2_O; synthesis temperature 90 °C. Hereafter, the hydrotalcite layer involved in the studies was synthesized under the above-mentioned optimal conditions.

The hydrotalcite layer synthesis solution was prepared as follows: a salt solution was first prepared by dissolving certain quantities of magnesium chloride hexahydrate and aluminum nitrate in deionized water under vigorous stirring, and then the solution was mixed with sodium carbonate aqueous solution under agitation. The pH of the solution was adjusted with 0.1 mol L^−1^ sodium hydroxide. The synthesis solution was vigorously stirred for 6 h before using it for the synthesis of the hydrotalcite layer.

The support was vertically placed into the autoclave, which had been charged with the above-prepared hydrotalcite synthesis solution. Then, the reactor was sealed and placed into the drying oven, which has been heated to 90 °C, and kept for 24 h. After that, the tube was taken out from the hydrotalcite synthesis solution when the reactor was cooled to room temperature, and repeatedly washed with deionized water until the pH of the washed water was neutral. Finally, the obtained tube was dried at 110 °C for 12 h before the subsequent synthesis of NaA zeolite membrane.

### 2.3 Preparation of NaA zeolite membrane

The zeolite membrane synthesis solution was prepared *via* a similar procedure reported elsewhere.^24^ Briefly, sodium silicate solution was first prepared by dissolving sodium hydroxide in a colloidal silica solution under heating and stirring, then, it was cooled to room temperature and mixed with sodium aluminate aqueous solution under agitation. The synthesis solution was vigorously stirred for 24 h before using it for the synthesis of NaA zeolite membrane.

NaA zeolite membranes were synthesized by the microwave heating method. Firstly, the supports were vertically placed into the Teflon reactor, which had been charged with the above-prepared zeolite membrane synthesis solution. After that, the reactor was placed into a microwave oven, which was connected with a reflux device. The synthesis solution was raised to 90 °C in 5 min and maintained for 30 min under ambient pressure using microwave power. After the synthesis solution was cooled to room temperature, the tube was taken out from the zeolite membrane synthesis solution and washed with deionized water repeatedly until the pH of the washed water was neutral. Finally, the thus obtained tube was dried at 110 °C for 12 h before subsequent characterization.

The detailed information of the synthesis condition is shown in Table 1.

**Table 1:** Synthesis conditions for the zeolite membranes

Sample	Hydrotalcite layer	Reaction composition of the zeolite membrane
M0	Yes	None
M1	None	2Na_2_O : 2.0SiO_2_ : Al_2_O_3_ : 200H_2_O
M2	Yes	2Na_2_O : 2.0SiO_2_ : Al_2_O_3_ : 200H_2_O
M3	Yes	2Na_2_O : 1.8SiO_2_ : Al_2_O_3_ : 200H_2_O
M4	Yes	2Na_2_O : 2.2SiO_2_ : Al_2_O_3_ : 200H_2_O
M5	Yes	2Na_2_O : 2.4SiO_2_ : Al_2_O_3_ : 200H_2_O
M6	Yes	1Na_2_O : 2.0SiO_2_ : Al_2_O_3_ : 200H_2_O
M7	Yes	3Na_2_O : 2.0SiO_2_ : Al_2_O_3_ : 200H_2_O
M8	Yes	4Na_2_O : 2.0SiO_2_ : Al_2_O_3_ : 200H_2_O

### 2.4 Characterization

X-ray diffraction (XRD) studies were conducted using Bruker D8 Advance X-ray diffraction equipment. The crystal structure of the membrane inside the tubes was hard to detect owing to the small curvature radius of the tubes. As a substitute, the powders were deposited in the reactor during the membrane preparation process were collected for XRD studies.

Scanning electron microscopy (SEM) was performed using an S4800 scanning electron microscope and the elemental content of the membranes was analyzed using energy-dispersive X-ray spectroscopy (EDX). The tube fragment with the outer smooth surface was selected for scanning electron microscopy studies.

Atomic Force Microscopy (AFM) was performed using a MultiMode 8 atomic force microscope. The tube fragment with the outer smooth surface was selected for atomic force microscopy studies.

### 2.5 Evaluation of gas permeation

The evaluation of permeation performance of the membranes was studied using a self-designed equipment, which was similar to that reported in the literature.^25^ The permeation flux *J* (mol m^−2^ s^−1^ Pa^−1^) and permselectivity α were calculated using eqn (1) and (2), respectively:1
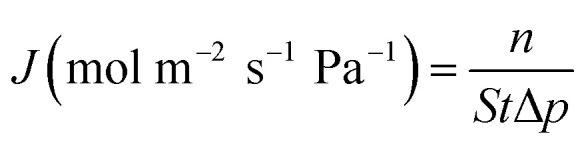
2
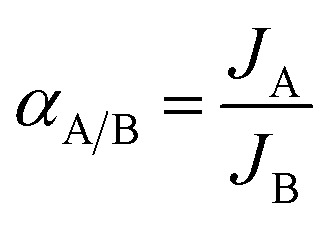
*n* – moles of permeated gas; Δ*p* – pressure difference between the two sides of zeolite membrane; *S* – the permeating area of zeolite membrane; *t* – permeating time; A and B – the determined gases.

## 3. Results and discussion

### 3.1 Effects of hydrotalcite layer on the zeolite membranes

**Fig. 1:**
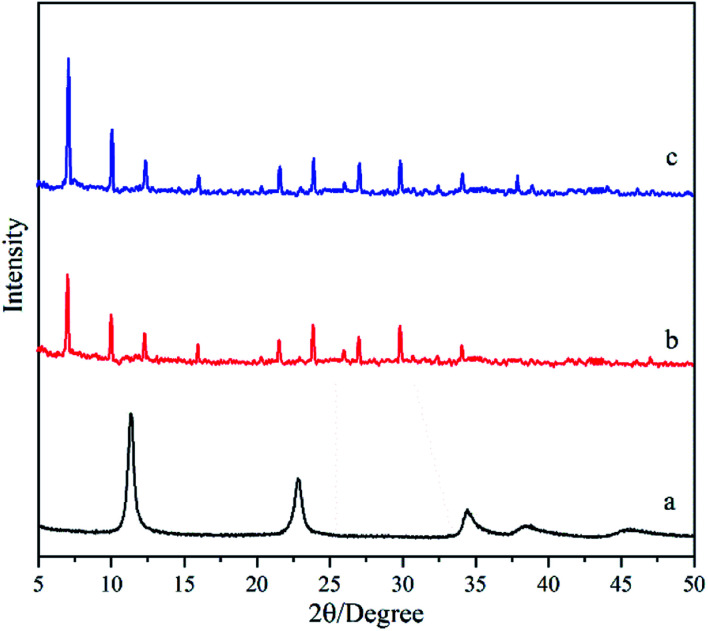
XRD patterns of the deposited powders obtained under different treatments: (a) M0, (b) M1, and (c) M2 membranes.

Fig. 1 shows XRD patterns of the deposited powders obtained under different treatments. It was found that the XRD pattern of the M0 membrane (Fig. 1a) exhibits obvious diffraction peaks at 2*θ* = 11.33°, 22.84° and 34.74°, agreeing well with the standard peaks of hydrotalcite crystal (JCPDS # 35-0965). It indicates that pure-phase hydrotalcite crystals were grown from the hydrotalcite synthesis solution during the *in situ* hydrothermal synthesis method at 90 °C for 24 h. The XRD patterns of M1 membrane (Fig. 1b) and M2 membrane (Fig. 1c) exhibit similar peak positions and peak intensities, both of which are well consistent with those of the NaA zeolite crystal (JCPDS # 39-0223). It suggests that active ingredients in the zeolite membrane synthesis solution can be transferred into NaA zeolite by the microwave heating method at 90 °C for 30 min, and the hydrotalcite layer on the surface of the support does not introduce any impure phases during the zeolite membrane synthesis process.

**Fig. 2:**
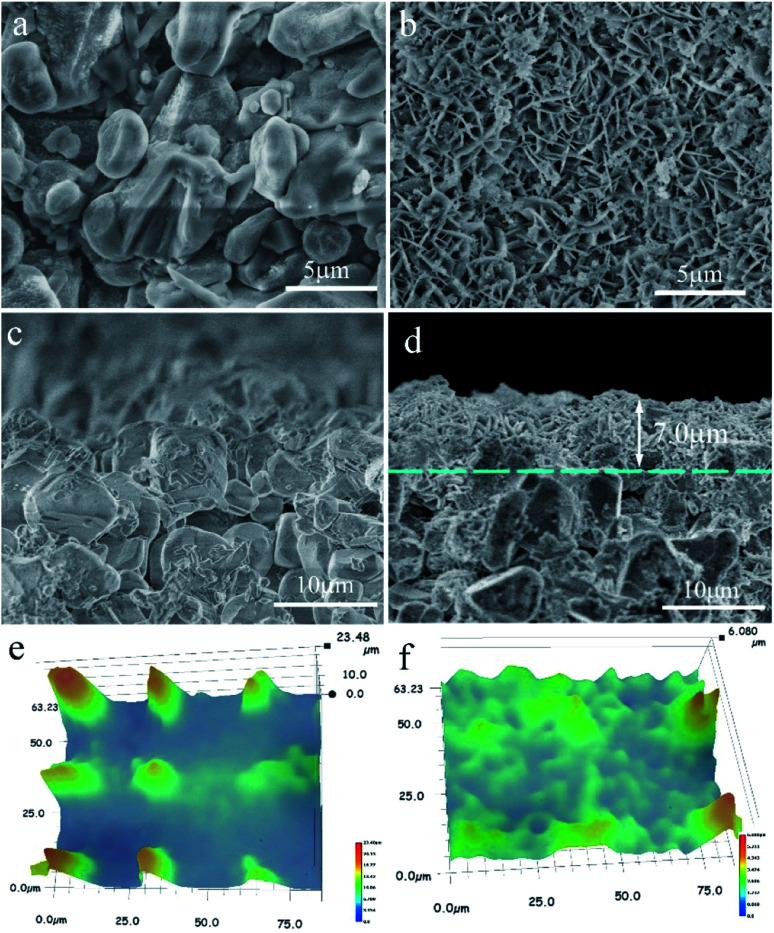
Micrographs of the support and the hydrotalcite interlayer. SEM micrographs: top-view of (a) support and (b) M0 membranes; cross-section of (c) support and (d) M0 membranes; AFM micrographs of (e) support and (f) M0 membranes.

Fig. 2 shows the micrographs of the support and the hydrotalcite interlayer. The support is sintered with the heterogeneous α-Al_2_O_3_ particles, while the pores in the support are formed from the gaps between the sintered particles (Fig. 2a and c). The heterogeneous α-Al_2_O_3_ particles make the surface of the support full of bumps and holes (Fig. 2e), which may lead to the difficult uniform distribution of the zeolite crystal on the surface of the support. After the support is hydrothermally treated in the hydrotalcite synthesis solution, flaky hydrotalcite crystals with a diameter of several microns and thickness of several nanometers grow vertically on the surface of the support, and they cross each other to form a reticulated hydrotalcite layer (Fig. 2b). The cross-sectional view (Fig. 2d) shows that a reticulated hydrotalcite layer with thickness of 7.0 μm is grown over the support, and exposes a flat and uniform surface. The uneven surface of the support is filled with hydrotalcite, leading to no clear cracks existing between the hydrotalcite layer and the support. The surface becomes smooth and flat (Fig. 2f), indicating that the hydrotalcite layer greatly improves the smoothness and regularity of the surface.

**Fig. 3:**
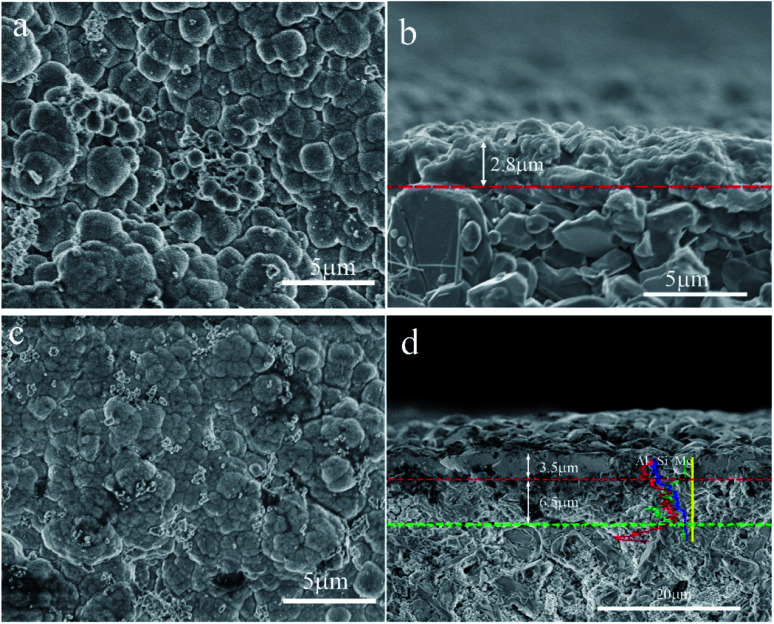
SEM micrographs of the zeolite membranes: top-view of (a) M1 and (c) M2 membranes; cross-section of (b) M1 and (d) M2 membranes.

Fig. 3 shows the SEM micrographs of the zeolite membranes. A layer of NaA zeolite with spherical morphology is formed on the surface of the support after the support is treated under microwave heating in the zeolite membrane synthesis solution (Fig. 3a and c), which agrees with the reports on microwave synthesis of zeolite membrane.^26,27^ The rough support makes the zeolite crystal unable to stack tightly and the zeolite layer uneven, resulting in large numbers of defects in the surface of the M1 membrane (Fig. 3a). In contrast, zeolite crystals are closely combined in the form of twins to form a defect-free zeolite layer in the M2 membrane (Fig. 3c) owing to the existence of the even reticulated hydrotalcite layer in the support, which agrees with the previous report.^28^ The cross-sectional view (Fig. 3b and d) shows that the zeolite layer with a thickness of 2.8 μm is grown over the support. However, some α-Al_2_O_3_ particles are exposed to the surface of the zeolite layer, leading to defects in the M1 membrane. The M2 membrane consists of support, hydrotalcite interlayer, and zeolite layer (Fig. 3d). The zeolite layer with a thickness of 3.5 μm is grown over the hydrotalcite layer and there is no clear crack existing between the zeolite layer and the hydrotalcite layer, indicating a strong interaction between them. The line EDS results show that the content of the elements is distinctly different between the different layers. There is a transition layer between each layer in the M2 membrane, indicating a strong combination between the layers. It may be due to the fact that the Si/Al active ingredients are deposited in the reticulate structure of the hydrotalcite layer and transferred into zeolite crystal, leading to the zeolite crystal growing in the reticulate structure and finally the reticulate structure effectively improves the stability of the zeolite layer. The partial dissolution of the hydrotalcite under strong alkali conditions results in a decrease in the thickness of the hydrotalcite layer.

A pencil hardness test was performed to further evaluate the mechanical strength of the zeolite membrane as reported elsewhere.^29,30^ The pencil is tilted to 45° on the zeolite membrane with a pressure force of 7.5 N. While some of the zeolite particles are broken when the 4H pencil streaks across the zeolite layer, the zeolite layer is not damaged, indicating that the zeolite layer can resist the 4H pencil hardness test. However, some scratches appear in the zeolite layer when the 5H pencil streaks across the zeolite layer, indicating that the hardness greater than that of the 4H pencils can damage the zeolite layer.

Accordingly, the hydrotalcite interlayer not only can improve the flatness and integrity of the support, limiting the formation of defects, but also can control the infiltration of the active ingredients into the surface of support using its reticulated structure, improving the stability of the top layer with the bottom layer.

### 3.2 Effects of the ratio of Si/Al on the zeolite membranes

**Fig. 4:**
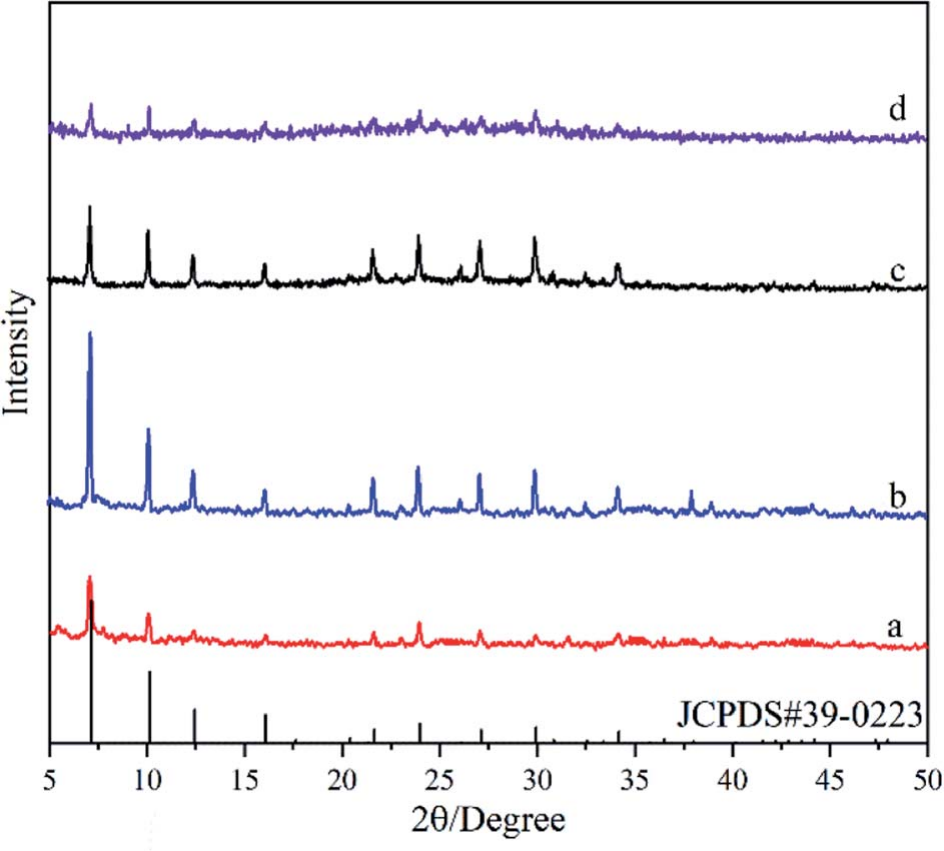
XRD patterns of the deposited powders obtained under different Si/Al ratios from the synthesis solution: (a) M3 membrane, (b) M2 membrane, (c) M4 membrane, (d) M5 membrane.

Fig. 4 shows XRD patterns of the deposited powders obtained under different Si/Al ratios from the synthesis solution. It was found that diffraction peaks in XRD patterns for all powders are consistent with the standard peaks of NaA zeolite crystal (JCPDS # 39-0223), indicating the formation of NaA zeolite crystals. The peak intensity first increases and then decreases with the ratio of Si/Al, and it reaches the maximum in the M2 membrane. It should be addressed that a broad peak from 20° to 30° appeared in the XRD pattern of the M5 membrane, indicating that the crystallinity of the NaA zeolite crystal is not high and some amorphous materials are still present. The XRD results suggest that the ratio of Si/Al can influence the formation of zeolite crystals, and too high or too low Si/Al ratio is not conducive to the growth of NaA zeolite crystals (the Si/Al ratio of the standard NaA crystal is 1 : 1), which agrees with that reported elsewhere.^31^

**Fig. 5:**
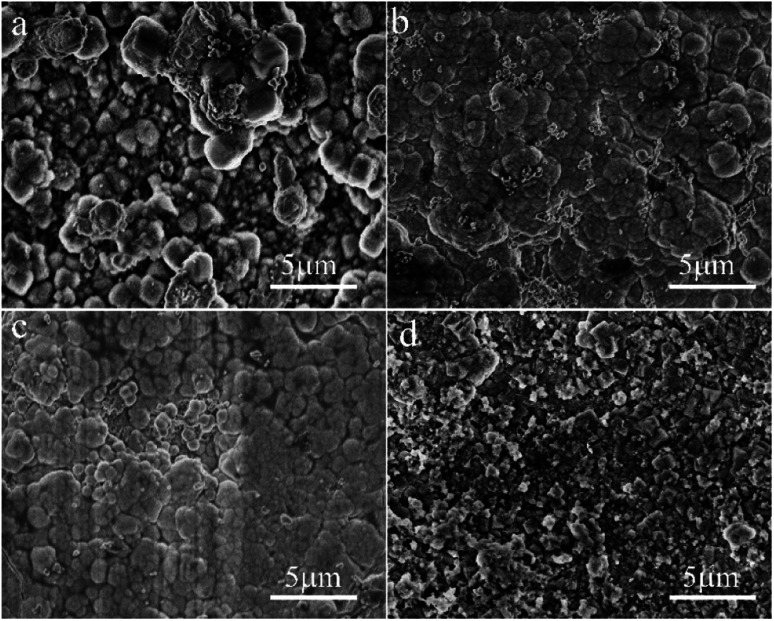
SEM micrographs of the top view of the support treated with different Si/Al ratios from the synthesis solution: (a) M3 membrane, (b) M2 membrane, (c) M4 membrane, (d) M5 membrane.

Fig. 5 shows SEM micrographs of the top view of supports treated with synthesis solutions of different Si/Al ratios. One can see that a continuous solid-particle layer was formed over all supports even though the morphologies of the solid particles were different. The spherical grains with uneven particle size are loosely aggregated over the surface of the support for the M3 membrane (Fig. 5a), causing the construction of the zeolite layer with many defects. The membrane becomes denser as the ratio of Si/Al increases, and almost no obvious defects can be identified in the M2 and M4 membranes (Fig. 5b and c). When the ratio of Si/Al in the synthesis solution far exceeds the standard Si/Al ratio of NaA zeolite (the Si/Al ratio of the standard NaA crystal is 1 : 1), fine solid particles with different morphology is deposited on the surface of the support, forming a solid particle layer with many defects (Fig. 5d). The SEM results show that the ratio of Si/Al can significantly influence the formation of zeolite crystals and the Si/Al ratio of the synthesis solution for the M2 membrane can be considered as the best condition, which is similar to the XRD results.

### 3.3 Effects of alkalinity on zeolite membranes

**Fig. 6:**
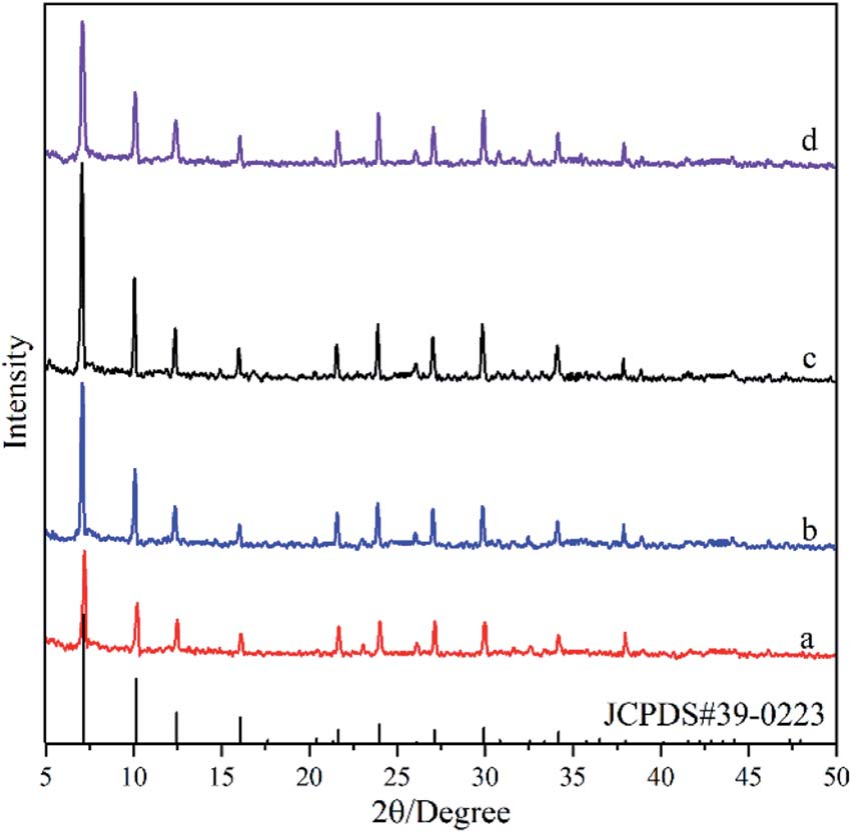
XRD patterns of the deposited powders obtained under different alkalinities in the synthesis solution: (a) M6 membrane, (b) M2 membrane, (c) M7 membrane, (d) M8 membrane.

Fig. 6 shows the XRD pattern of the deposited powders obtained under different alkalinities in the synthesis solution. It was found that the diffraction peaks in XRD patterns for all the powders are consistent with the standard peaks of NaA zeolite crystals (JCPDS # 39-0223), even though their intensities are different, indicating the formation of NaA zeolite crystals. The peak intensities in the XRD pattern of the M6 membrane are the lowest, indicating that the low alkalinity in the synthesis solution is not conducive to the growth of NaA zeolite. The peak intensity increases and then decreases with alkalinity, and it reaches the maximum in the M7 membrane. It can be due to the fact that the low alkalinity is not enough to dissolve the components in the synthesis solution, which reduces the crystallinity of the NaA zeolite. The increase in the alkalinity can not only increase the concentration of the components in the synthesis solution but also can increase the crystallization rate of NaA zeolite crystals, leading to the increasing intensity of the XRD patterns.^32^ However, when the alkalinity is too high, the hydroxyl group would hinder the growth of the zeolite crystals that have the negative charge, leading to decreasing intensity of XRD patterns when the alkalinity is too high.^33^

**Fig. 7:**
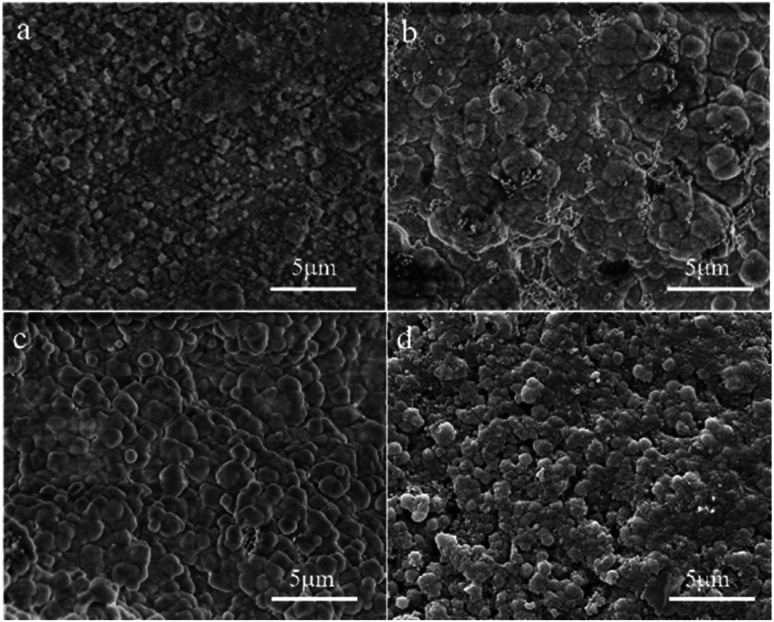
SEM micrographs of the top view of the ceramic tube treated at different alkalinities in the synthesis solution: (a) M6 membrane, (b) M2 membrane, (c) M7 membrane, (d) M8 membrane.

Fig. 7 shows SEM micrographs of the top view of supports treated under different alkalinities in the synthesis solution. One can see that a continuous solid particle layer has been formed over all supports even though the morphologies of the solid particles are different. The fine spherical grains are aggregated over the surface of the support for the M6 membrane (Fig. 7a). However, there are lots of defects in the zeolite layer due to insufficient contact between the spherical grains. The particle size of the spherical grains increases gradually with the increase in alkalinity, and they contact each other in the form of twins, leading to the quality of the membranes becoming denser with the increase in alkalinity. The surface of the M7 membrane (Fig. 7c) is the smoothest with almost no obvious defects, indicating that the zeolite layer is complete and compact. When the alkalinity is so high, pinhole-like defects are formed in the zeolite layer, which affects the compactness of the zeolite membrane. It can be due to the fact that the high alkalinity leads to the dissolution of some zeolite crystals, leaving holes to form defects. The SEM results show that the alkalinity in the synthesis solution can influence the quality of the zeolite membrane and the alkalinity of the synthesis solution for the M7 membrane can be considered the best condition, and the SEM data are similar to the XRD results.

### 3.4 Gas permeation performance of the zeolite membranes

In order to detect the densification of the membranes, the permeation performance of H_2_ and N_2_ through various membranes and the corresponding permselectivities of H_2_ over N_2_ was evaluated and the data are shown in Table 2. One can see that the permeance fluxes of H_2_ are higher than that of N_2_ for all membranes. The permeance flux of H_2_ through the support was 5.326 × 10^−5^ mol m^−2^ s^−1^ Pa^−1^, and its permselectivity for H_2_ over N_2_ was 2.27, which is much lower than the corresponding Knudsen diffusion coefficient of 3.74. When the hydrotalcite layer grows on the surface of the support (M0 membrane), both the permeance flux and permselectivity are similar to that for the support. It can be due to the fact that the flaky hydrotalcite crystals grow vertically on the surface of the support and they cross with each other to form large grid holes. When the gases pass through the reticulated hydrotalcite layer, the mass transfer resistance is small, but there is little selectivity. After the growth of the zeolite membrane on the surface of the hydrotalcite layer (the M2 membrane), the permeance flux of H_2_ decreases to 2.32 × 10^−6^ mol m^−2^ s^−1^ Pa^−1^, which is less than one the tenth of that of the M0 membrane. While its permselectivity of H_2_ over N_2_ is increased from 2.58 to 3.93, indicating that the M2 membrane has good compactness. In comparison, the permselectivity of H_2_ over N_2_ for the M1 membrane, the zeolite layer of which grows on the surface of the support directly, is only 3.18, which is lower than the corresponding Knudsen diffusion coefficient. The gas permeation performances suggest that the reticulated hydrotalcite layer is helpful in the preparation of dense zeolite membrane, which is similar to XRD and SEM results. The permeation of H_2_ through the zeolite membrane synthesized under the optimal conditions (the M7 membrane) achieved as high as 0.47 × 10^−6^ mol m^−2^ s^−1^ Pa^−1^, and its permselectivity for H_2_ over N_2_ was 4.70. The zeolite membrane has good stability for long-term separation operation (shown in Fig. S4†). The gas permeation properties of the NaA zeolite reported in the literature show that the fluxes of H_2_ are on the order of 10^−6^ to 10^−7^ mol m^−2^ s^−1^ Pa^−1^, which agree with this report. The permselectivity of the M7 membrane is similar to that of the zeolite membranes on tube support prepared using the microwave heating method. Although the permselectivity of the membranes is lower than that prepared using the conventional methods, this method greatly shortens the synthesis time, and supports used in this work are as long as 170 mm, which are substantially longer than those reported in the literature.

**Table 2:** Gas permeation performance of the zeolite membranes

Membrane	Support/length	Synthesis method	H_2_ permeance flux (10^−6^ mol m^−2^ s^−1^ Pa^−1^)	*α*(H_2_/N_2_)	Reference	Knudsen diffusion coefficient
Support	Tube/170 mm	Microwave	53.26	2.27	This work	3.74
M0	Tube/170 mm	Microwave	38.91	2.58	This work	
M1	Tube/170 mm	Microwave	14.33	3.18	This work	
M2	Tube/170 mm	Microwave	2.32	3.93	This work	
M7	Tube/170 mm	Microwave	0.47	4.70	This work	
NaA	Tube/70 mm	Microwave	0.24	4.71	34	
NaA	Disks	Microwave	2.36	4.14	35	
NaA	Disks	Microwave	3.65	5.62	36	
NaA	Tube/—	Conventional	0.528	6.66	17	
NaA	Disks	Conventional	0.23	6.02	37	

## 4. Conclusion

A novel strategy for preparing compact zeolite membranes on rough support with the assistance of a reticulated hydrotalcite layer was developed in this study. The reticulated hydrotalcite layer was grown on the inner surface of the 170 mm length ceramic tube by an *in situ* hydrothermal method, and then the NaA zeolite membrane was prepared on this reticulated layer by a microwave heating method. The effects of the reticulated hydrotalcite layer, the ratio of Si/Al, and alkalinity were investigated in detail. The results show that the hydrotalcite interlayer not only improved the flatness and integrity of the support, limiting the formation of defects, but also controlled the infiltration of the active ingredients into the surface of support using its reticulated structure, improving the stability of the zeolite layer. The optimal molar ratio of the synthesis solution for the synthesis of zeolite membrane was 3Na_2_O : 2SiO_2_ : Al_2_O_3_ : 200H_2_O. The permeance flux of H_2_ through the zeolite membrane synthesized under the optimal conditions achieved as high as 0.47 × 10^−6^ mol m^−2^ s^−1^ Pa^−1^, and its permselectivity for H_2_ over N_2_ was 4.7, which was higher than the corresponding Knudsen diffusion coefficient.

## Conflicts of interest

There are no conflicts to declare.

## Supplementary Material

RA-011-D1RA05132F-s001
