# Supplementary material for: In situ constructed zeolite membranes on rough supports with the assistance of reticulated hydrotalcite interlayer
Source: RSC Adv. 2021 Nov 17;11(59):37131–7. doi: 10.1039/d1ra05132f (PMC9043786; doi:10.1039/d1ra05132f)
Supplement: RA-011-D1RA05132F-s001 [file RA-011-D1RA05132F-s001.pdf]

Electronic Supplementary Material (ESI) for RSC Advances.
This journal is © The Royal Society of Chemistry 2021

Support Information

In-situ construct zeolite membranes on rough support with the assisting of reticulated hydrotalcite interlayer

Wen-Yan Pan, Liang-Liang Peng, Wen-Jing Wang, Yuan-Yuan Li, Xue-Ling Wei*

School of Chemical and Environmental Engineering, Anhui Polytechnic University, Wuhu, Anhui 241000, China

**Corresponding authors:*

PhD. Xue-Ling Wei;

E-mail: xueling_wei@163.com.

1. Effect of alkalinity on the hydrotalcite interlayer

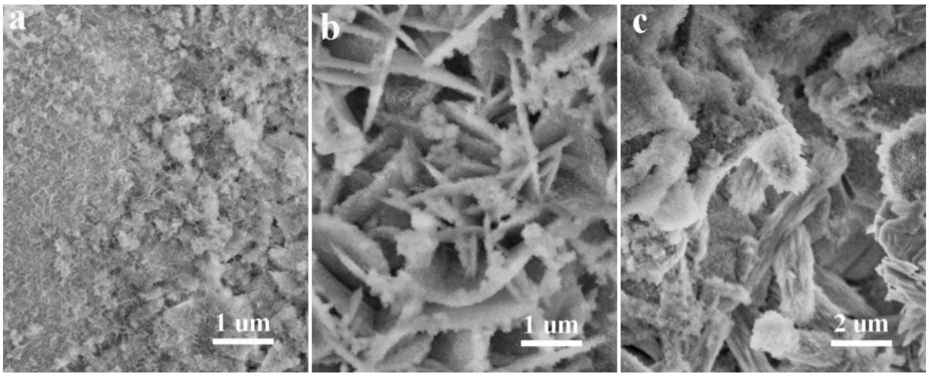

Fig. S1 SEM micrographs of hydrotalcite interlayer synthesized of different alkalinities. The molar compositions of the synthesis solution were 6 $MgCl_2 \cdot 6H_2O$：2 $Al(NO_3)_3 \cdot 9H_2O$：x NaOH：1 Na_2CO_3：666 H_2O, where x=(a) 10, (b) 20 and (c) 30. The hydrotalcite interlayer were prepared at 90 °C for 24h.

Fig. S1 shows the SEM micrographs of hydrotalcite interlayer fabricated using synthesis solution with different alkalinities. When the alkalinity is low, there is only a small amount of flake structure with a large number of fine particles on the surface of the support, indicating that the low alkalinity is not conducive to the growth of the hydrotalcite crystals. When the content of NaOH is 20, flaky hydrotalcite crystals with diameter of several microns and thickness of several nanometers grow vertically on the surface of the support, and they cross each other to form a reticulated hydrotalcite layer (Fig. S1b). However, when the alkalinity is high, some rod structures are formed on the surface of the support even the network structures also can be detected. It can be due to the fact that the high alkalinity will accelerate the dissolution and recrystallization of the hydrotalcite and destroy the network structure of the interlayer. Accordingly, the optimal Na_2O content in the synthesis solution is 20.

2. Effect of Mg/Al ratio on the hydrotalcite interlayer

Fig. S2 shows the SEM micrographs of hydrotalcite interlayer fabricated using synthesis solution with different Mg/Al ratios. When the Mg/Al ratio is 3:1 and 2:1, the sheeted hydrotalcite crystals crosslink into a network structure forming a reticulated hydrotalcite interlayer on the surface of the support, while, there is only small sheet structures on the surface of the support when the Mg/Al ratio is 1:1. There are lots of defects in the sheeted hydrotalcite crystal when the Mg/Al ratio is 2:1,

leading to the fragile reticular structure of the hydrotalcite interlayer. It can be due to the fact that the low Mg/Al ratio in the synthesis cannot provide sufficient Mg for the growth of hydrotalcite (The standard Mg/Al ratio in hydrotalcite is 3:1), resulting in incomplete growth of the sheeted structure. Accordingly, the optimal Mg/Al ratio in the synthesis solution is 3:1.

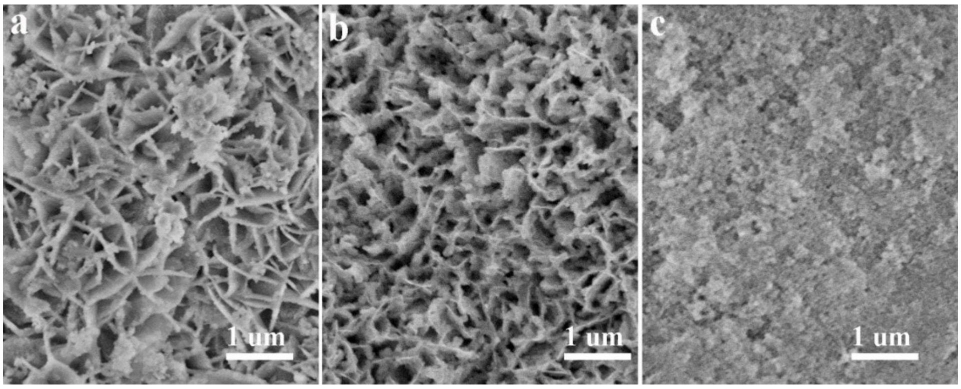

Fig. S2 SEM micrographs of hydrotalcite interlayer synthesized of different Mg/Al ratios. The molar compositions of the synthesis solution were x $MgCl_2 \cdot 6H_2O$: 2 $Al(NO_3)_3 \cdot 9H_2O$: 20 NaOH: 1 Na_2CO_3: 666 H_2O, where x=(a) 6, (b) 4 and (c) 2. (d) is the enlarged view of (c). The hydrotalcite interlayer were prepared at 90 °C for 24h.

3. Effect of synthesis temperature on the hydrotalcite interlayer

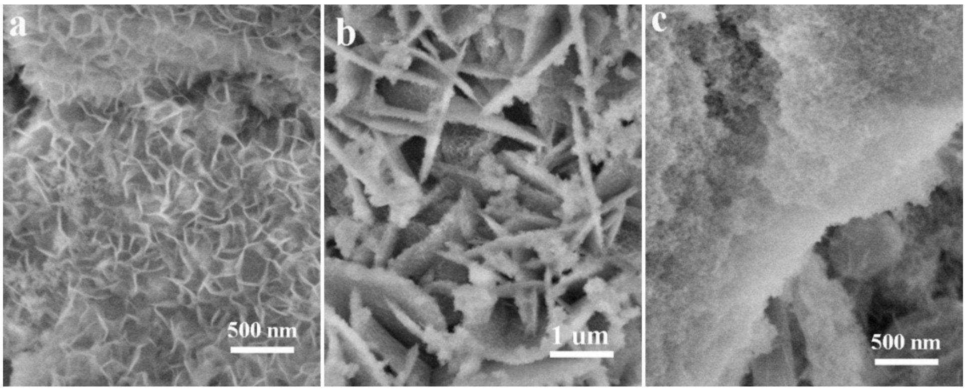

Fig. S3 SEM micrographs of hydrotalcite interlayer synthesized using different synthesis temperatures: (a) 80 °C, (b) 90 °C, (c) 100 °C, and (d) 150 °C. The molar compositions of the synthesis solution were 6 $MgCl_2 \cdot 6H_2O$: 2 $Al(NO_3)_3 \cdot 9H_2O$: 20 NaOH: 1 Na_2CO_3: 666 H_2O; The hydrotalcite interlayer were prepared at different temperatures for 24h.

Fig S3 shows the SEM micrographs of the hydrotalcite interlayer fabricated using different synthesis temperatures. When the synthesis temperature is 80 °C, the hydrotalcite interlayer formed by the interconnection of the nano-sheeted hydrotalcite, however, the size of the hydrotalcite is too small to from the continuous reticulated

structure. when the synthesis temperature is 90 °C, the hydrotalcite sheet and their reticular structure are fully grown to form a continuous hydrotalcite interlayer. When the synthesis temperature rises to 100 °C, the growth rate of hydrotalcite accelerates, and the nano-sheeted hydrotalcite crystals grow rapidly to form block structure. Accordingly, the optimal synthesis temperature is 90 °C.

It is obtained that the optimized synthesis conditions are as follows: synthesis sol molar composition 6 $MgCl_2{\cdot}6H_2O$： 2 $Al(NO_3)_3{\cdot}9H_2O$： 20 NaOH： 1 Na_2CO_3： 666 H_2O; synthesis temperature 90 °C.

4. Stability of the zeolite membranes

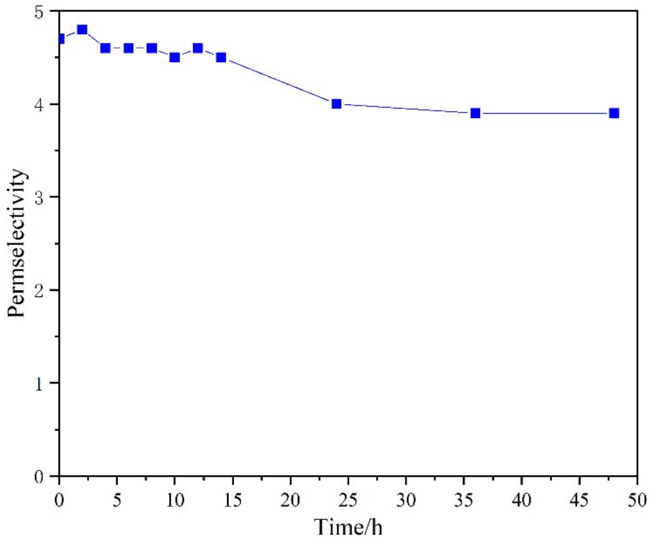

Fig. S4 The stability of the M7 membrane

To test the stability of the zeolite membrane, N2 was continuously injected into the M7 membrane and tested its separation performance at certain intervals. The permselectivity stability of the M7 membrane was shown in Fig. S4. The permselectivity of the M7 membrane fluctuated around 4.6 in the first ten hours, indicating that the M7 membrane had good stability. However, if the operation time was too long, the permselectivity decreased significantly and gradually approached the Knudsen diffusion coefficient. It may be due to the fact that the water in the gas was adsorbed into the zeolite crystal and the hindered the passage of the gas. The separation performance of the zeolite membrane was restored after heating.
